# Regulation of Hyaluronan Synthesis in Vascular Diseases and Diabetes

**DOI:** 10.1155/2015/167283

**Published:** 2015-03-05

**Authors:** Paola Moretto, Evgenia Karousou, Manuela Viola, Ilaria Caon, Maria Luisa D'Angelo, Giancarlo De Luca, Alberto Passi, Davide Vigetti

**Affiliations:** Department of Surgical and Morphological Sciences, University of Insubria, 21100 Varese, Italy

## Abstract

Cell microenvironment has a critical role determining cell fate and modulating cell responses to injuries. Hyaluronan (HA) is a ubiquitous extracellular matrix glycosaminoglycan that can be considered a signaling molecule. In fact, interacting with several cell surface receptors can deeply shape cell behavior. In vascular biology, HA triggers smooth muscle cells (SMCs) dedifferentiation which contributes to vessel wall thickening. Furthermore, HA is able to modulate inflammation by altering the adhesive properties of endothelial cells. In hyperglycemic conditions, HA accumulates in vessels and can contribute to the diabetic complications at micro- and macrovasculature. Due to the pivotal role in favoring atherogenesis and neointima formation after injuries, HA could be a new target for cardiovascular pathologies. This review will focus on the recent findings regarding the regulation of HA synthesis in human vascular SMCs. In particular, the effects of the intracellular HA substrates availability, adenosine monophosphate-activated protein kinase (AMPK), and protein O-GlcNAcylation on the main HA synthetic enzyme (i.e., HAS2) will be discussed.

## 1. Introduction

Cardiovascular pathologies are the major cause of death in western countries, and their impact is increasing due to rising rates of obesity and diabetes [1]. Diabetes is the most widespread metabolic disorder and its medical and socioeconomic burden is caused by the associated complications that are mostly at macrovascular and microvascular level, leading to retinopathy, neuropathy, and nephropathy, as a consequence of accelerated atherogenesis [2, 3]. Limited success of pharmacological and invasive-surgical (i.e., angioplasty and bypass grafting) treatments may be a result of the incomplete understanding of the biological mechanisms which control and contribute to the development of atherosclerosis. At biochemical level, during hyperglycemic conditions, several alterations have been described in different pathways as polyol, hexosamine, protein kinase C, and advanced glycation end-product (AGE) metabolisms [2].

The development of atherosclerosis is coupled to dramatic alterations of the extracellular matrix (ECM), which provides critical support for vascular tissue acting as a scaffold for maintaining the organization of vascular cells into blood vessels, for blood vessel stabilization, and for cell proliferation, migration and survival [4–6]. ECM is a complex milieu of macromolecules that influences the activities of the cells, including cell differentiation, migration, and proliferation by specific cell-matrix interactions [7]. Hyaluronan (HA) is a ubiquitous ECM component with a multitude of functions [8]. HA is a linear polymer belonging to the family of glycosaminoglycans (GAGs), which comprises the major fraction of carbohydrates in ECM. HA is present in low amounts in normal blood vessels but increases dramatically in vascular diseases [9–11].

In this review, we will discuss the new regulatory mechanisms that link HA synthesis, atherosclerosis, and diabetes.

## 2. Hyaluronan

HA is a linear GAG that is composed of repeating units of D-glucuronic acid (GlcUA) and N-acetylglucosamine (GlcNAc) linked together through alternating *β*-1,4 and *β*-1,3 glycosidic bonds. This disaccharide can be repeated several thousand times without any other chemical modification (i.e., sulfation, acetylation, and epimerization) that are typical of the other GAGs [12]. Differently from the other GAGs, HA is not covalently bound to any core protein of proteoglycans, although HA can interact with other ECM molecules as versican, aggrecan, and tumor necrosis factor- (TNF-) stimulated gene 6 (TSG-6) via particular domains (i.e., link domain) [13]. HA is a very multifunctional GAG and HA properties and effects on cells depend on the length of the polysaccharide chains. In tissues, HA molecular mass can range from 500,000 to 10,000,000 Da [13].

HA appeared late during evolution and it is present only from chordate, probably with the aim of modulating the immune system and cells motility [14, 15]. Interestingly, some pathological bacteria (i.e.,* Streptococcus equisimilis*,* Streptococcus pyogenes*, and* Pasteurella multocida*) possess the operon that permits both the synthesis of precursors and HA polymerization. This HA stealth or capsule makes the bacteria not easily identifiable by antibodies or attacked by phagocytes.

HA has been considered a mere space filling molecule for a long time, able to modulate tissue hydration. More recently, HA was shown to have other peculiar properties. For instance, high molecular weight HA has typically anti-inflammatory and antiangiogenic properties and inhibits cell proliferation. On the other hand, low molecular weight HA shows opposite characteristics, favoring inflammation and promoting cell growth [16]. These effects are often mediated by several cell surface receptors, including CD44, receptor for HA-mediated motility (RHAMM), lymphatic vessel endothelial receptor 1 (Lyve-1), HA receptor for endocytosis (HARE), and Toll-like receptors 4 and 2 (TLR4-2), all of them able to trigger different intracellular signaling cascades [17]. Moreover, chemical modifications of HA with TSG-6 and bikunin alter the properties of high molecular weight HA [18].

At least three different mechanisms are known to produce low molecular weight HA. High molecular weight HA fragmentation can be achieved either by chemical agents, as free radicals and oxidative stress [19], or by the action of specific degrading enzymes (i.e., hyaluronidases) that chop HA in the extracellular space and, further, continue the degradative process inside the cells [20]. The third mechanism involves the synthetic process. Normally, cells synthesize high molecular weight HA, but metabolic alterations or dysfunctions in the synthetic enzymes could influence the length of the polysaccharide.

HA synthesis is catalyzed by a family of three HA synthases (HAS1, HAS2, and HAS3) that are multipass transmembrane enzymes. HASes use cytosolic UDP-GlcUA and UDP-GlcNAc and are able to extrude the nascent polysaccharide chain through the plasma membrane into the ECM [21]. These HAS isoenzymes have different kinetic properties; in fact HAS3 produces shorter HA chains (ranging from <2 × 10^5^ Da to 3 × 10^5^ Da) with respect to HAS1 and HAS2 that synthesize larger polymers (up to 2 × 10^6^ Da) [22, 23]. An extremely high molecular mass HA of about 12 MDa is produced by naked mole rats (*Heterocephalus glaber*), which display exceptional longevity, with a maximum lifespan exceeding 30 years [24]. This very long HA protects naked mole rat from tumors and is produced by a HAS2 enzyme with critical substitutions in the catalytic domain [24]. HAS2 is also the predominant isoform in mammals and HAS2 knockout mice die early in gestation due to heart defects, whereas HAS1 or HAS3 null mice are normal and fertile [25, 26]. Recently, in dermal fibroblasts, HAS1 was found to be activated by hyperglycemic conditions and by proinflammatory cytokines [27], suggesting a role during nutrients abundance.

**Figure 1:**
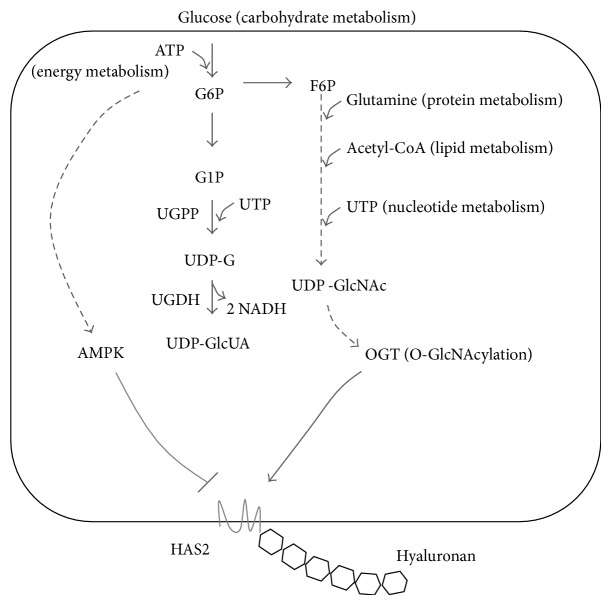
HA precursors biosynthesis and main HAS2 regulation in SMCs. Glucose enters in the cells and is phosphorylated by ATP. Glucose 6 phosphate (G6P) can be converted into glucose 1 phosphate (G1P), UDP-glucose (UDP-G), and UDP-glucuronic acid (UDP-GlcUA) by the enzymatic reactions catalyzed by UDP-G pyrophosphorylase (UGPP) and UDP-G dehydrogenase (UGDH). This latter reaction produces 2 NADH. G6P can enter in the hexosamine biosynthetic pathway, which starts from fructose 6 phosphate (F6P) and, in several steps, produces UDP-N-acetylglucosamine (UDP-GlcNAc). These steps depend on carbohydrates, energy, proteins, lipids, and nucleotides metabolisms making UDP-GlcNAc a master nutrient sensor. AMPK in condition of ATP depletion (or activation by metformin) inhibits HAS2 by threonine 110 phosphorylation. An increment of UDP-GlcNAc induces O-GlcNAcylation of serine 221 of HAS2 by OGT. This glycosylation strongly stabilizes HAS2 protein avoiding its degradation.

UDP-sugar precursors of HA synthesis are produced in the cytoplasm by two different pathways (Figure 1) [28]. UDP-GlcUA derives from glucose-1-phosphate which is linked to UDP forming UDP-glucose in the irreversible reaction catalyzed by UDP-glucose pyrophosphorylase. UDP-glucose is then oxidized to UDP-GlcUA by the peculiar enzyme UDP-glucose dehydrogenase that catalyzes the double oxidation of the C6 hydroxyl group in the carboxylic group forming two NADH. UDP-GlcNAc can be formed starting from glucose or by glucosamine through the hexosamine biosynthetic pathway (Figure 1).

It is noteworthy that cytoplasmic concentration of UDP-sugars can fluctuate in function of synthetic enzymatic activities and nutrients availability (i.e., glucose) [2, 28, 29]. Therefore, HASes can work using saturating or subsaturating concentration of substrates. This can greatly influence the length of the secreted polysaccharides, as previously demonstrated using purified bacterial HAS [30, 31]. In contrast to HA, the other GAGs are synthesized inside the Golgi apparatus and the high affinity UDP-sugar transporters ensure a high concentration of precursors independently from nutrients availability [32].

## 3. Role of HA and ECM in Vascular Diseases

Vascular diseases are pathological conditions of arteries that are triggered by endothelial cell dysfunction. Because of factors like pathogens, oxidized LDL particles, and other inflammatory stimuli, endothelial cells become activated and start to synthesize proinflammatory molecules (i.e., cytokines and chemokines) and express adhesion molecules on their surface. This enhances the recruitment of circulating immune cells (i.e., monocytes and lymphocytes) that infiltrate in the vessel wall. Because of endothelial cytokines and immune cell infiltration, SMCs start to proliferate and migrate towards the blood vessel lumen. Moreover, SMCs secrete several ECM molecules (i.e., HA and versican) and EMC degrading enzymes (i.e., matrix metalloproteinases) leading to the thickening of the vessel wall. Atherosclerotic plaque consists of proliferating SMCs, macrophages, and various types of lymphocytes that can obstruct blood flow, leading to diminished amounts of oxygen and nutrients to the surrounding tissues. Eventually, plaque may also rupture causing the formation of clots [33, 34].

HA and the proteoglycan versican are greatly involved in vascular remodeling [11, 35]. Versican is a proteoglycan that interacts with HA forming large aggregates within the blood vessels ECM. Via several domains, versican can mediate binding to cytokines, enzymes (like ADAMTS4), lipoproteins, other extracellular matrix molecules, and signaling receptors [36, 37]. HA/versican are increased in human restenotic lesions that are formed after balloon angioplasty, in pseudoaneurysms of the human temporal artery, in advanced human atherosclerotic plaques, and in plaque thrombus interface, suggesting possible roles in the thrombotic processes [38, 39]. Other proteoglycans are known to modulate vascular ECM as the small leucine-rich repeat proteoglycan biglycan, decorin, and osteoglycin [40–42] even if these molecules do not directly interact with HA.

Vessel thickening is associated with proliferating, migrating, and dedifferentiated arterial SMCs, suggesting a role for these ECM molecules in controlling smooth muscle behavior [43]. Interestingly, also endothelial cells can synthesize HA after proinflammatory stimuli, altering adhesive capacity and recruiting of immune cells [44, 45]. The critical proatherosclerotic properties of HA are demonstrated in several manners. Transgenic HAS2 mice showed an accelerated neointima formation after injury [46] whereas the inhibition of HA synthesis (by using 4-methylumbelliferone) reduced neointima formation [47].* In vitro* experiments, 4-methylumbelliferone blocked SMC proliferation, migration, and induced apoptosis [48]. Moreover, the rescuing with high molecular weight HA restored cell viability by inhibiting cell death [49]. CD44 knockout mice, lacking the main HA receptor, were protected against atherosclerosis [50].

As aging is one of the major risk factors for the insurgence of vascular pathologies [51], it is not surprising that many works report the augment of HA content in aged vessels [52–56] and that senescent human SMCs enhance HA synthesis* in vitro* [57].

Although the causes of atherosclerosis are still debated, the critical role of oxidized low density lipoproteins (ox-LDL) is well accepted [58]. SMCs treated with oxLDL, but not modified LDL, dramatically induced HA secretion* in vitro* as well as cell proliferation and migration. Interestingly, the blocking of scavenger receptor LOX-1 [59] reduced HA synthesis and inhibits cell migration [60].

These evidences indicate the role of HA in promoting atherosclerosis. A better understanding of the regulatory mechanisms of its production could be useful to limit HA synthesis in order to counteract vessel thickening.

## 4. HA Synthesis Regulation by Substrates

One of the major points of regulation of HA synthesis is on HASes [61]. First of all, HASes have to reach the plasma membrane and, therefore, are synthesized as part of the secretory pathway. What happens to HASes proteins during ER and Golgi trafficking is not known but it is known that they can be active in intracellular vesicles [62, 63]. This can explain the presence of intracellular HA that seems unrelated to lysosomal turnover [64]. Proinflammatory cytokines increase HASes activity in intracellular compartments leading to the formation of particular filamentous HA structures called HA cables [62]. These cables that emerge from perinuclear structures have the capability to efficiently bind immune cells contributing to inflammation [65, 66] and therefore it could be of great importance to correlate these cables with TSG6-bikunin modified HA [18].

The availability of precursors is also important for controlling HA synthesis since UDP-glucose pyrophosphorylase and dehydrogenase are known to be necessary for sustaining HA production [28]. Although these two enzymes have critical functions in glycogen biosynthesis and in detoxification, little is known about their regulation. In aged SMCs, the increased HA secretion is associated with high levels of both UDP-glucose dehydrogenase and HASes mRNAs [57]. Interestingly, the other GAGs seem not influenced by UDP-GlcUA availability. Therefore, HASes and UDP glucose dehydrogenase could be regulated in a similar manner.

The other HA precursor, UDP-GlcNAc, is the most abundant UDP-sugar within the cells and its concentration greatly depends on the nutrients availability [29]. In fact, hexosamine biosynthetic pathway integrates carbohydrates, lipids, amino acids, and nucleotides metabolisms and is considered one of the most important nutrient sensors in the cells [67]. HA synthesis is influenced by UDP-GlcNAc in at least three aspects. The first regards the substrate availability as all GAGs seem to be altered by UDP-GlcNAc [68]. UDP-GlcNAc controls UDP-N-acetylgalactosamine availability by the action of the UDP-galactose 4-epimerase enzyme [69]. In this way, UPD-GlcNAc regulates also GAGs containing N-acetylgalactosamine.

Secondly, UDP-GlcNAc concentration regulates the activity of the O-GlcNAc transferase (OGT) [29]. OGT is the critical enzyme that catalyzes the transfer of the UDP-GlcNAc to serine or threonine residues of nucleocytoplasmic proteins. This intracellular glycosylation is named O-GlcNAcylation [70]. Although OGT can be regulated posttranslationally [71], this enzyme possesses low affinity for its substrate [72]. Therefore, only when UDP-GlcNAc increases, OGT starts to modify proteins by O-GlcNAcylation. Many critical proteins are regulated by O-GlcNAcylation and HAS2 is among them [68]. O-GlcNAcylation greatly stabilizes HAS2 in the membrane, leading to an increased HA synthesis. Interestingly, as OGT is a nucleocytoplasmic protein, O-GlcNAcylation regulates only HA synthesis without affecting other GAGs synthetic enzymes in the Golgi.

**Figure 2:**
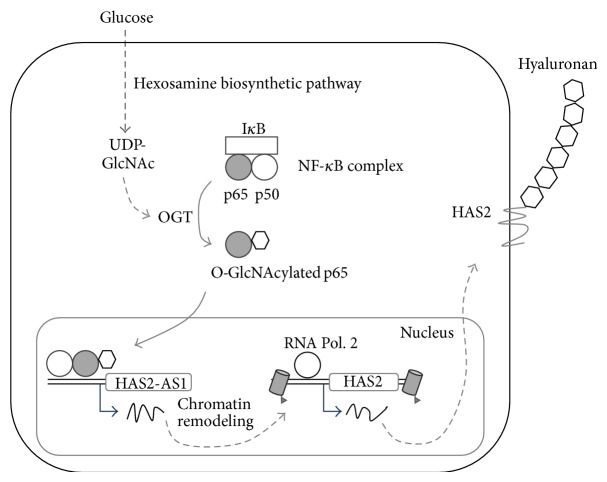
Nuclear control of HAS2 transcription by O-GlcNAcylation. In condition of glucose abundance, OGT modifies p65 by means of O-GlcNAcylation. Glycosylated p65 induces the transcription of HAS2-AS1 that, in turn, enhances HAS2 transcription. The mechanism through which HAS2-AS1 drives HAS2 transcription is complex and partially unknown. Natural antisense RNA can bind enzymes involved in epigenetic modifications. Recent evidences highlight that HAS2-AS1 is able to open chromatin structure around HAS2 promoter enhancing RNA polymerase 2 and other factors accessibility.

Thirdly, UDP-GlcNAc controls HAS2 expression via OGT, NF-*κ*B, and HAS2-AS1 [73]. The latter is the natural antisense transcript (a particular type of long noncoding RNA) for HAS2 transcribed using the opposite strand of HAS2 locus on chromosome 8. HAS2 and HAS2-AS1 RNA molecules share about 200 base pairs and can form RNA:RNA duplex that stabilizes HAS2 transcript and favors HA synthesis [74]. However, RNA stabilization is not involved in the increase of HAS2 expression due to UDP-GlcNAc augment. Recent findings revealed that OGT triggers HAS2-AS1 transcription which, in turn, is necessary to enhance HAS2 transcription (Figure 2) [73]. As long noncoding RNAs modulate epigenetic modifications, such as acetylation and methylation [75], HAS2-AS1 could represent a new element able to regulate HA synthesis via epigenetic modifications. Interestingly, NF-*κ*B subunit p65 is associated to HAS2-AS1 promoter but not to HAS2 promoter, suggesting the critical role of such noncoding RNA in the regulation of inflammatory properties of HA [73].

## 5. AMPK and HA

Metabolism has a crucial role to control HA synthesis via substrate availability while a special role is played by cell energy content [28]. HA is a very high energy consuming molecule. The synthesis of an averaged size HA chain, which contains ten thousand disaccharides, represents considerable energy expenditure for the cell. To form a single chain, almost fifty thousand ATP equivalents, twenty thousand NAD cofactors, and ten thousand acetyl-CoA groups are required, in addition to the monosaccharide components and amino groups [76].

Adenosine monophosphate-activated protein kinase (AMPK) has a pivotal role in regulating energy homeostasis in eukaryotic cells [77]. In response to a decrease in cellular ATP levels, AMPK leads to a reduction in the rate of anabolic pathways (ATP-utilizing) and an increase in the rate of catabolic pathways (ATP-producing) [78]. This regulation is due to the phosphorylation of several key enzymes, including HAS2 [79].

In response to low ATP, AMPK inhibits specifically HA synthesis in vascular SMCs [79]. The phosphorylation of HAS2 threonine 110 blocks the HA synthetic process, whereas HAS1 and HAS3 are not AMPK substrates [79]. AMPK activation is known to protect from neointima formation [80, 81] and one of the mechanisms* in vivo* could be HA synthesis inhibition.

## 6. HA and Diabetes

Macro- and microangiopathies are the main complications of diabetes. Because of the tight connection between metabolism and HA synthesis, it is possible that HA and diabetes are linked. In serum of diabetic patients, HA amounts and HA staining in vessels are known to be elevated [82, 83]. Similar results were found in a porcine model of diabetes [84] and in SMCs grown in high glucose medium (mimicking diabetes) [85]. Also, nephropathies are associated with diabetes. Indeed, rat mesangial cells are known to increase HA production in hyperglycemic conditions and recruit immune cells in a HA-dependent manner [86–88]. Interestingly, recent evidences found that HA is involved in inflammation of pancreatic islets, highlighting a potential role for HA in the pathogenesis of type 1 diabetes [89, 90].

HA is also involved in diabetic ulcers favoring the healing process [91]. Diabetic foot makes up 50% of all nontraumatic amputations [92]. Peripheral neuropathy and vascular disease are thought to be major factors causing chronic foot ulcerations [93]. The use of HA or of engineered HA scaffolds (mainly composed of HA benzyl esters) with cultured expanded autologous fibroblasts and keratinocytes enhance the healing process by supporting cells proliferation and migration but also providing tissue hydration [94].

From a biochemical point of view, there are several manners in which the enhanced glucose availability induces HA synthesis. Although the effects on HA synthesis of AGEs are not known, it is known that such compounds can induce fragmentation of high molecular weight HA [95], favoring a proinflammatory response via TLR4-2. Moreover, it is also known that high molecular weight HA protects against the proinflammatory effects of AGEs [96].

Protein kinase C (PKC) isoforms dependent on diacylglycerol are known to be activated in cultured microvascular cells of diabetic animals [2]. This is due to the increased levels of DAG in hyperglycemic conditions. As it is well known that PKC activators enhanced HA synthesis [97], it is clear that in diabetic conditions PKC is a plausible cause of HA accumulation [63].

In hyperglycemic conditions, the excess of glucose is known to enter in the hexosamine biosynthetic pathway, leading to an increase of UDP-GlcNAc [29]. As discussed above, this induces a strong HA synthesis activation, as well as the alteration of HAS2 expression [68, 73]. O-GlcNAcylation is also increased in hyperglycemia [98]. Moreover, several proteins (i.e., HAS2 and endothelial nitric oxide synthase) [99] and transcription factors (SP1 and YY1) are regulated by this type of posttranslational modification [100].

Diabetes insurgence depends on a variety of factors while nutrients and lifestyle have a crucial role. High-fat diet is known to be linked with type 2 diabetes [101, 102] and recently it was discovered that rodents fed a high-fat diet leaded to accumulation of HA in skeletal muscle, which contributes to insulin resistance [103].

Nutrients can alter gene expression through epigenetics [104]. Epigenetics plays a critical role in both type 1 and type 2 diabetes [105] and can be involved in the so-called “metabolic memory” [106, 107]. Metabolic memory theory foresees that early hyperglycemic environment is remembered in the target organs (i.e., eye, kidney, heart, blood vessels, and extremities) via epigenetic modifications and that such modifications could persist for years also during positive antidiabetic therapies. As the incidence of diabetic complications is not directly linked to the blood glucose concentration [108], metabolic memory could have a critical role in this issue. As HA and other ECM components synthesis can be controlled by epigenetic modification [109], cell microenvironment could be critical for metabolic memory effects.

Moreover, AMPK is strictly related to diabetes [77, 110]. Although it is not so clear whether metformin, a well-known hypoglycemic drug [111, 112], directly or indirectly activates AMPK, it is known that it specifically reduces the synthesis of HA in SMCs [79] and hyperinsulinemia [113]. Although still debated, metformin could have vasoprotective and antitumoral effects [113–115], which could derive from reducing HA production.

## 7. Conclusions

ECM remodeling is emerging to have a pivotal role in several pathologies contributing to vascular diseases onset and progression. HA can have a multitude of effects on the vascular cells behavior. Several new mechanisms are recently discovered to regulate HA metabolism, all of them linked to glucose availability. A deeper understanding of such mechanisms will permit the identification of potential new pharmacological targets for the treatment of vascular pathologies.

## Abbreviations

AGE: Advanced glycation end-products
ECM: Extracellular matrix
HA: Hyaluronan
SMCs: Smooth muscle cells
UDP-GlcNAc: Uridine diphosphate N-acetylglucosamine
HAS: Hyaluronan synthase
AMPK: Adenosine monophosphate activated protein kinase
OGT: O-GlcNAc transferase
oxLDL: Oxidized low density lipoproteins
RHAMM: Receptor for hyaluronan-mediated motility
HARE: Hyaluronan receptor for endocytosis
TLR: Toll-like receptor
PKC: Protein kinase C.
